# Autotetraploidization Gives Rise to Differential Gene Expression in Response to Saline Stress in Rice

**DOI:** 10.3390/plants11223114

**Published:** 2022-11-15

**Authors:** Ningning Wang, Shiyan Wang, Fan Qi, Yingkai Wang, Yujie Lin, Yiming Zhou, Weilong Meng, Chunying Zhang, Yunpeng Wang, Jian Ma

**Affiliations:** 1 Faculty of Agronomy, Jilin Agricultural University, Changchun 130117, China; 2 Institute of Agricultural Biotechnology, Jilin Academy of Agricultural Sciences, Changchun 130033, China

**Keywords:** diploid, autotetraploid, saline stress, phytohormones, gene expression

## Abstract

Plant polyploidization represents an effective means for plants to perpetuate their adaptive advantage in the face of environmental variation. Numerous studies have identified differential responsiveness to environmental cues between polyploids and their related diploids, and polyploids might better adapt to changing environments. However, the mechanism that underlies polyploidization contribution during abiotic stress remains hitherto obscure and needs more comprehensive assessment. In this study, we profile morphological and physiological characteristics, and genome-wide gene expression between an autotetraploid rice and its diploid donor plant following saline stress. The results show that the autotetraploid rice is more tolerant to saline stress than its diploid precursor. The physiological characteristics were rapidly responsive to saline stress in the first 24 h, during which the elevations in sodium ion, superoxide dismutase, peroxidase, and 1-aminocyclopropane-1-carboxylic acid were all significantly higher in the autotetraploid than in the diploid rice. Meanwhile, the genome-wide gene expression analysis revealed that the genes related to ionic transport, peroxidase activity, and phytohormone metabolism were differentially expressed in a significant manner between the autotetraploid and the diploid rice in response to saline stress. These findings support the hypothesis that diverse mechanisms exist between the autotetraploid rice and its diploid donor plant in response to saline stress, providing vital information for improving our understanding on the enhanced performance of polyploid plants in response to salt stress.

## 1. Introduction

Whole-genome duplication (WGD) is a significant and frequently occurring event in plant evolution [1]. The polyploidization supports multiple sets of genetic materials for natural adaptation. Usually, polyploid plants are dichotomized into two categories by virtue of the origin of the additional genomes: autopolyploid and allopolyploid [2]. The former typically arise from chromosome doubling of a single diploid genome, whereas an allopolyploid is formed through interspecific hybridization. The dramatic increase in the size of the plant genome leads to phenotypic variation, genome instability, chromosome imbalance, regulatory incompatibility, and reproductive failures. However, due to genetic diversity, polyploid plants are able to better adapt and withstand a wider range of environmental stresses than their diploid precursors, featuring larger vegetative organs, additional secondary metabolites and greater stress resistance [2,3,4,5]. As Haldane hypothesized more than 90 years ago [6], in the short-term, polyploidization could greatly reduce the effect of genetic load by masking recessive or partial-recessive deleterious mutations due to increased allelic multiplicity. As a result, it has been postulated that polyploidization caused by whole-genome duplication can temporarily improve fitness, although there is a lack of sufficient empirical data to support such a supposition. Even more so, population genetic theory predicts both short-term and long-term benefits of polyploidization, and emerging empirical data suggests that established polyploids may act as “sponges” accumulating adaptive allelic diversity. It appears that polyploidization can provide instantaneous fitness benefits, paving the way for selective breeding.

It is well established that allopolyploidization plays a vital role in supporting plant diversification through both hybridization and polyploidization, yet the utilization of autopolyploidization is hindered by the frequent occurrence of meiotic instability and subsequent dwindling in fertility [7,8]. Nevertheless, numerous studies have shown that autopolyploids have a greater ability to tolerate stress than their diploid counterparts [9,10]. There is a broad set of factors that interact with each other to alter the response of autopolyploid cells to particular abiotic stress factors. The gained adaptability in polyploid plants, however, is thought to stem from biochemical and physiological changes [11]. Salt tolerance in Arabidopsis (*Arabidopsis thaliana*) has been substantially improved in merely one generation since autotetraploidization, as compared to isogenic diploids. The neo-autotetraploid A. *thaliana* lines were shown to experience a fitness tradeoff, being less fit under non-saline conditions, but better adapted after being subjected to a saline challenge, relative to their diploid progenitors [12]. In autotetraploid cassava, the alteration in anatomy led to stronger drought tolerance [13]. Likewise, autotetraploid rice exhibited a stronger tolerance to short periods of exposure to alkaline stress [14]. More evidence of the molecular events that underlie the observed improvement in abiotic stress tolerance was found by capitalizing on the recent development of deep-sequencing. According to a number of studies [12,15,16], the amelioration in susceptibility to abiotic stress has been linked to polyploidization in plants.

Soil saline-alkalization, as a typical abiotic stress factor that limits crop production, is becoming an increasingly serious problem worldwide [17,18]. Salt-alkali stress can be categorized into mild (the salt content < 3‰, the pH value ~ 7.1–8.5), moderate (~3–6‰, 8.5–9.5), and severe (>6‰, >9.5) types based on the salt content and pH value [19]. It is generally recognized that saline stress and alkaline stress are two distinct types of abiotic stress for plants, in spite of their common co-existence, in which the former is mainly caused by neutral salt [20,21]. The saline stress can induce osmotic stress, ionic toxicity, and nutritional depletion in plants [22,23]. The unbalanced ions are exchanged by plant roots and transported to shoots by transpiration, impeding plant growth and negatively affecting crop quality [24,25].

Plants suffering from salt-alkali stress often display symptoms of the sodium ion (Na^+^) toxicity, which widely impact plant growth and development. Ionic stress is caused by the ingress of Na^+^ into the cell, while osmotic stress is caused by a high concentration of ions that drives water molecules out of the cell [18]. Because of the high concentration of Na^+^ in saline stress simulators such as NaCl, Na_2_SO_4_, and other neutral salts, plants are deprived of essential nutrients, such as potassium ion (K^+^) [18]. To maintain a constant intracellular and intercellular osmotic potential, cells produce and store a variety of small molecule organic compounds, such as proline, soluble proteins, and soluble sugars [26]. Meanwhile, in response to osmotic stress, reactive oxygen species (ROS) will accumulate, resulting in severe oxidative damage to cell structure [27,28]. Thus, antioxidant enzymes and antioxidants, such as superoxide dismutase (SOD), catalase (CAT), and peroxidase (POD), will increase to alleviate ROS stress in plants [18,26]. Malondialdehyde (MDA) generated from lipid peroxidation will be scavenged by these enzymes to safeguard the membrane structure [18].

Phytohormones play a crucial role in plant growth and development. Endogenous phytohormones, such as abscisic acid (ABA), can respond rapidly to salinity and osmotic stress and regulate plant transpiration by controlling stomatal opening and closing [29,30], and modulating ROS concentration by regulating calcium ion (Ca^2+^) channels in guard cells [31,32]. In response to saline stress, ABA interacts with ethylene (ET), auxin (such as indole acetic acid, IAA), cytokinin (CK), salicylic acid (SA), and jasmonic acid (JA). Arabidopsis plants treated with 1-aminocyclopropane-1-carboxylic acid (ACC), a precursor to ethylene, displayed improved salt tolerance across a range of developmental stages [33,34]. Similarly, IAA mediation in root growth may facilitate an adaptive response to salt stress in plants [35,36]. A negative response to salt stress in plants, mediated by CK (trans-zeatin (tZ) and isopentenyladenine), was elucidated [37,38]. The increase in JA levels was found to be associated with hyper sensitivity to salinity in tomatoes [39] and rice [40]. In addition, in response to saline stress, SA has been reported to promote plant growth and contribute to robust plant defense systems in a dose-dependent manner [41,42]. In response, the genes involved in phytohormone metabolism and signal transduction have been found to be expressed differentially in response to saline stress [18,42,43]. There is, however, a lack of coordinated investigation on genome-wide transcriptome and metabolomics in response to saline stress by plants of various ploidy levels, which is crucial for understanding the relationship between salt resistance and ploidy levels.

In this study, we employed metabolome and transcriptome analyses to analyze the variations in phytohormone levels and gene transcription under saline stress to examine the short-term plant response to saline stress in an autotetraploid rice and its diploid donor. The primary goals of this study were: (1) to distinguish the morphological difference between autotetraploid rice and its diploid donor in response to saline stress; (2) to determine the differential effects of saline stress on Na^+^, K^+^, small molecule organic compounds, and phytohormone levels in diploid and autotetraploid rice; and (3) to shed light on the relevant genes associated with the regulation process of plant response to saline stress in a ploidy level-dependent manner.

## 2. Results

### 2.1. The Phenotypes and Ionic Content Were Altered Following Saline Stress in 9311-2x and 9311-4x Rice

**Figure 1:**
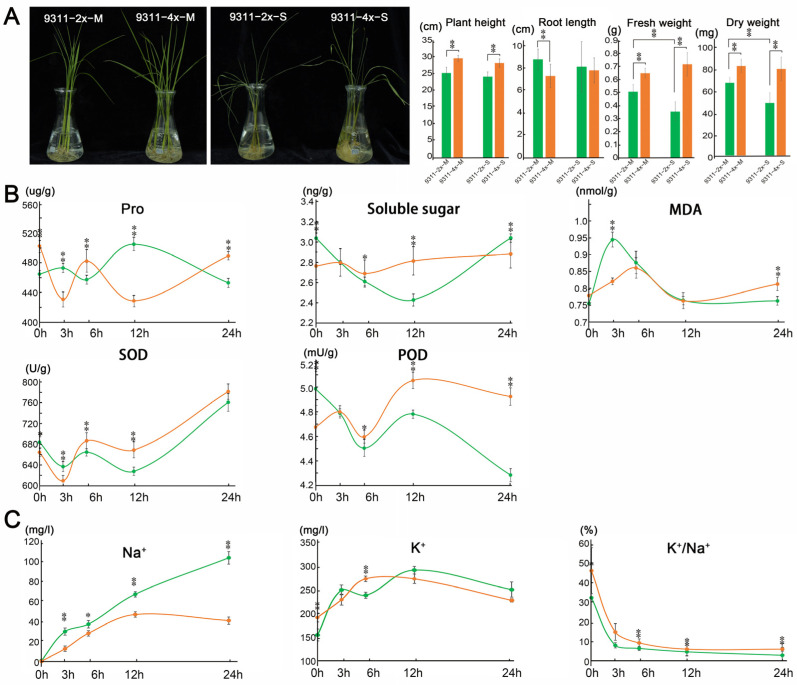
The morphological and physiological variations in 9311-4x and 9311-2x following saline stress. (**A**) The morphological variation in 9311-2x and 9311-4x following saline stress for 7 days (d). Plant image 9311-2x-M represents 9311-2x control, 9311-4X-M represents 9311-4x control, 9311-2x-S represents 9311-2x stressed for 7 d, 9311-4x-S represents 9311-4x stressed for 7 d. (**B**) Small molecule organic compounds variation in 9311-2x and 9311-4x following saline stress for 24 h. The orange and green lines represent salt-stressed 9311-4x and 9311-2x, respectively. (**C**) The concentration of Na^+^, K^+^, and the ratio variation of K^+^/Na^+^ in 9311-2x and 9311-4x following saline stress for 24 h. The orange and green lines represent 9311-4x and 9311-2x, respectively. ** *p* < 0.01, * *p* < 0.05.

The autotetraploid rice (*Oryza sativa*. ssp. Indica, 9311-4x) arose from chromosome doubling of the donor plant ‘Yangdao 6′ cultivar 93-11 (9311-2x), and the karyotypic analysis was described in our previous report [14]. Both 9311-2x and 9311-4x were subjected to NaCl solution (100 mM) treatment for 24 h (h) and 7 days (d). Significant morphological and phenotypic changes were observed in all the plants under saline stress for 7 d, including plant height, root length, fresh weight, and dry weight (Figure 1A). It is worthy of noting that the fresh and dry weights were significantly decreased following saline stress in 9311-2x, but not in 9311-4x, suggesting that autopolyploid plants were more tolerant to saline stress than their diploid counterparts. Such an observation is well in line with the study on Nipponbare rice [44].

However, phenotypic variation was not discernible following saline stress for 24 h. Ionic concentration was analyzed in rice plants following 24 h of exposure to saline stress in order to examine the potential variation in plant responses to short-term saline stress between these two types of rice plants (Figure 1B). It is evident that the Na^+^ concentration was significantly higher in 9311-2x than in 9311-4x at all the five time points within 24 h following saline treatment. On the other hand, the ratio of K^+^/Na^+^ was significantly higher in 9311-4x than in 9311-2x upon exposure to saline treatment for 6 h and 24 h. These results indicate that 9311-4x plants are more tolerant to saline treatment than 9311-2x, in light of ionic concentration measurement, within the first 24 h following saline stress.

After saline treatment for 24 h, the content of proline, soluble sugars and MDA, and the activities of two antioxidant enzymes, SOD and POD, were analyzed in both 9311-2x and 9311-4x plants (Figure 1C). Significant variations in most of these parameters were revealed between 9311-2x and 9311-4x at different timepoints of salt treatment. In particular, the extent of the increase in the activities of SOD and POD was significantly greater in 9311-4x than in 9311-2x following saline stress for 6 h and thereafter.

### 2.2. The Phytohormones Level Were Altered Following Saline Stress in 9311-2x and 9311-4x Rice

**Figure 2:**
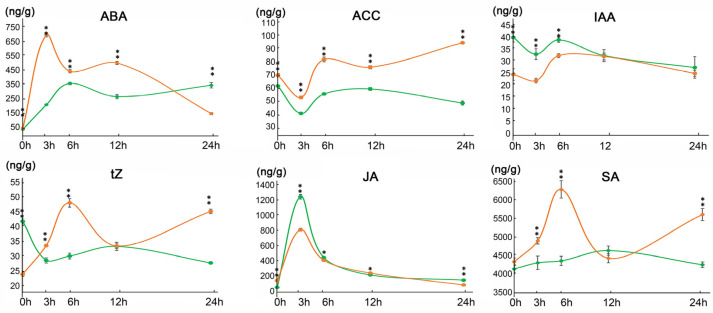
The variations in the content of phytohormones between 9311-4x and 9311-2x following saline stress for 24 h. The orange and green lines represent 9311-4x and 9311-2x, respectively. ** *p* < 0.01, * *p* < 0.05.

The concentration of a number of phytohormones, including ABA, ACC, IAA, tZ, JA and SA, was compared between 9311-2x and 9311-4x, following a period of saline stress treatment for 0, 3, 6, 12, and 24 h (Figure 2). It appears that most of these phytohormones exhibited significant variations between 9311-2x and 9311-4x at all the five timepoints of saline treatment. In particular, the ABA level was significantly higher than in 9311-4x than in 9311-2x in the first 12 h, but the opposite was true in the next 12 h of saline treatment. The ACC level was higher in 9311-4x than in 9311-2x at all the sampling time points. The IAA level was lower in 9311-4x than in 9311-2x within the first 6 h. The tZ level was higher in 9311-4x than in 9311-2x in the periods between 3 h to 6 h and between 12 h to 24 h. The JA level was higher in 9311-4x than in 9311-2x between 6 h and 12 h. The SA level was higher in 9311-4x than in 9311-2x within the first 6 h and in the period between 12 h and 24 h. It is apparent that significant variation in the amount of phytohormones exists between the autotetraploid rice and its diploid donor in response to saline stress.

### 2.3. The Genome-Wide Gene Expression Variation Induced by Saline Stress in 9311-2x and 9311-4x Rice

RNA-sequencing (RNA-seq) was used to profile gene expression in 9311-2x and 9311-4x following saline stress treatment for 6 h. Approximately 5G clean data were obtained for each sample, allowing for investigation of the potential differences in early response between diploid and autotetraploid rice (Table S1). The root samples from the 9311-2x plants and the 9311-4x plants were denoted MR2 and MR4, respectively. Root samples derived from the NaCl-treated plants of 9311-2x and 9311-4x were denoted SR2 and SR4, respectively. Likewise, MSh2 and MSh4 represent the shoot samples derived from 9311-2x and 9311-4x plants, respectively; SSh2 and SSh4 represent the NaCl-treated shoot samples derived from 9311-2x and 9311-4x plants, respectively.

**Figure 3:**
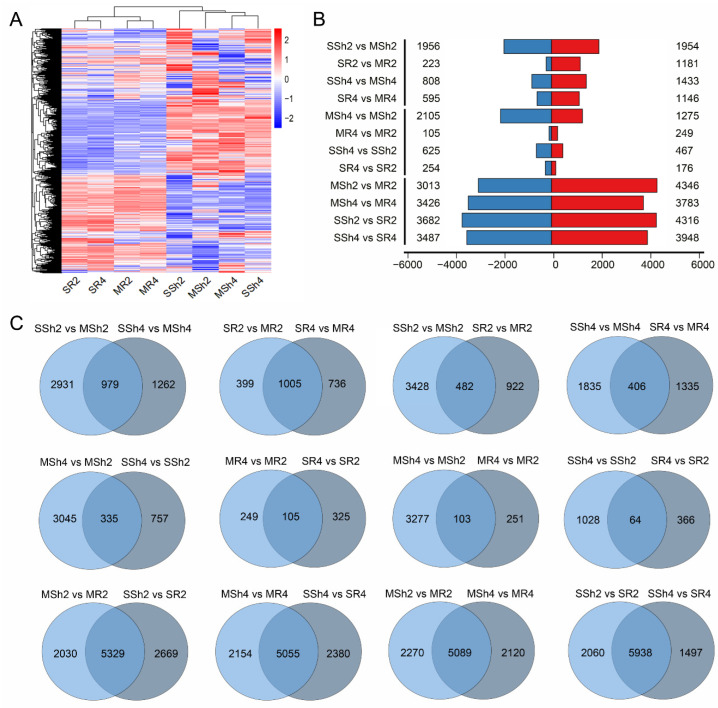
The transcriptome analysis in 9311-2x and 9311-4x following NaCl treatment for 6 h. (**A**) Heatmap of gene expression levels in shoots and roots. (**B**) The number of up-regulated (blue) and down-regulated (red) differentially expressed genes (DEGs) are marked at the side. (**C**) Details of the DEGs between all samples. MR2 and MR4 represent the root samples derived from 9311-2x and 9311-4x plants, respectively. SR2 and SR4 represent the root samples derived from NaCl-treated 9311-2x and 9311-4x plants, respectively. MSh2 and MSh4 represent the shoot samples derived from 9311-2x and 9311-4x plants, respectively. SSh2 and SSh4 represent the shoot samples derived from NaCl-treated 9311-2x and 9311-4x plants, respectively.

Roots of 9311-2x and 9311-4x rice expressed 18,390 and 18,553 genes under mock conditions (MR2 and MR4), and 18,593 and 18,398 genes under stressed conditions (SR2 and SR4), respectively (Figure 3A), whereas shoots of 9311-2x and 9311-4x rice expressed 19,324 and 18,430 genes under mock conditions (MSh2 and MSh4), and 19,138 and 18,629 genes under stressed conditions (SSh2 and SSh4), respectively. Figure 3B shows the number of expressed genes (DEGs) between 9311-2x and 9311-4x plants. In 9311-2x, there were 1954 up-regulated and 1956 down-regulated genes in SSh2 vs. MSh2, 1181 up-regulated and 223 down-regulated genes in SR2 vs. MR2. In 9311-4x, there were 1433 up-regulated and 808 down-regulated genes in SSh4 vs. MSh4, 1146 up-regulated and 595 down-regulated genes in SR4 vs. MR4. Moreover, there were 1275 up-regulated and 2105 down-regulated genes in MSh4 vs. MSh2; 2249 up-regulated and 105 down-regulated genes in MR4 vs. MR2. To compare saline stress and mock conditions between 9311-2x and 9311-4x, there were 467 up-regulated and 625 down-regulated genes in SSh4 vs. SSh2, 176 up-regulated and 254 down-regulated genes in SR4 vs. SR2. In addition, there were 4346 up-regulated and 3013 down-regulated genes in MSh2 vs. MR2, 3783 up-regulated and 3426 down-regulated genes in MSh4 vs. MR4. To compare saline stress and mock conditions between shoot and root, there were 4316 up-regulated and 3682 down-regulated genes in SSh2 vs. SR2, 3948 up-regulated and 3487 down-regulated genes in SSh4 vs. SR4.

The commonly or uniquely expressed genes in each sample were analyzed and presented in Figure 3C. (1) There were 2931 and 1262 uniquely expressed genes in SSh2 vs. MSh2, and SSh4 vs. MSh4, respectively. There were 979 commonly expressed genes in SSh2 vs. MSh2 and SSh4 vs. MSh4. There were 399 and 736 uniquely expressed genes in SR2 vs. MR2, and SR4 vs. MR4, respectively. There were 1005 commonly expressed genes in SR2 vs. MR2 and SR4 vs. MR4. For the comparison of NaCl treatment and Mock, there were 3428 and 922 uniquely expressed genes in SSh2 vs. MSh2, and SR2 vs. MR2, respectively; and 482 commonly expressed genes in SSh2 vs. MSh2 and SR2 vs. MR2. There were 1835 and 1335 uniquely expressed genes in SSh4 vs. MSh4, and SR4 vs. MR4, and 406 commonly expressed genes in SSh4 vs. MSh4 and SR4 vs. MR4. (2) For the comparison of 9311-2x and 9311-4x, there were 3045 and 757 uniquely expressed genes in MSh4 vs. MSh2, and SSh4 vs. SSh2, respectively; and 335 commonly expressed genes in MSh4 vs. MSh2 and SSh4 vs. SSh2. There were 249 and 326 uniquely expressed genes in MR4 vs. MR2, and SR4 vs. SR2, respectively; and 106 commonly expressed genes in MR4 vs. MR2 and SR4 vs. SR2. There were 3277 and 251 uniquely expressed genes in MSh4 vs. MSh2, and MR4 vs. MR2, respectively; and 103 commonly expressed genes in MSh4 vs. MSh2 and MR4 vs. MR2. There were 1028 and 366 uniquely expressed genes in SSh4 vs. SSh2, and SR4 vs. SR2, respectively; and 64 commonly expressed genes in SSh4 vs. SSh2 and SR4 vs. SR2. (3) For the comparison of shoot and root, there were 2030 and 2669 uniquely expressed genes in MSh2 vs. MR2, and SSh4 vs. SR2, respectively; and 5329 commonly expressed genes in MSh2 vs. MR2 and SSh2 vs. SR2. There were 2154 and 2380 uniquely expressed genes in MSh4 vs. MR4, and SSh4 vs. SR4, respectively; and 5055 commonly expressed genes in MSh4 vs. MR4 and SSh4 vs. SR4. There were 2270 and 2380 uniquely expressed genes in MSh2 vs. MR2, and SSh4 vs. SR4, respectively; and 5055 commonly expressed genes in MSh4 vs. MR4 and SSh4 vs. SR4. There were 2060 and 1497 uniquely expressed genes in SSh2 vs. SR2, and SSh4 vs. SR4, respectively; and 5938 commonly expressed genes in SSh2 vs. SR2 and SSh4 vs. SR4.

### 2.4. GO and KEGG Enrichment Analyses of DEGs between 9311-2x and 9311-4x Rice

**Figure 4:**
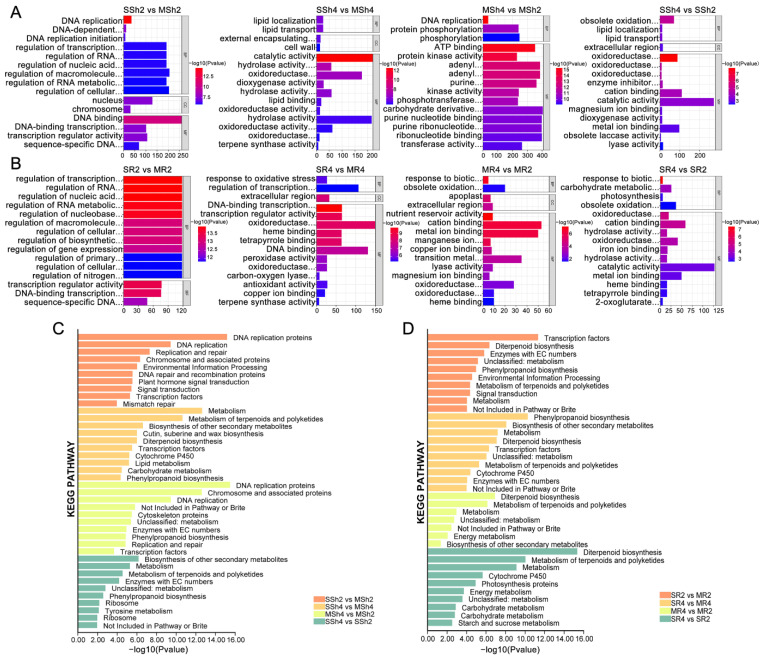
Gene ontology (GO) classification and enrichment analysis of the differentially expressed genes (DEGs) in shoots (**A**) and roots (**B**) between 9311-2x and 9311-4x. Kyoto encyclopedia of genes and genomes (KEGG) pathways of the significantly enriched DEGs in shoots (**C**) and roots (**D**) between 9311-2x and 9311-4x. MR2 and MR4 represent the root samples derived from 9311-2x and 9311-4x plants, respectively. SR2 and SR4 represent root samples derived from NaCl-treated 9311-2x and 9311-4x plants, respectively. MSh2 and MSh4 represent the shoot samples derived from 9311-2x and 9311-4x plants, respectively. SSh2 and SSh4 represent the shoot samples derived from NaCl-treated 9311-2x and 9311-4x plants, respectively.

The gene ontology (GO) enrichment analysis of the DEGs between various pairs of comparisons was conducted as shown in Figure 4A and Table S2 for shoots, in Figure 4B and Table S2 for roots. For SSh2 vs. MSh2, the top enriched GO terms for biological process (BP) category were DNA replication (GO:0006260), DNA-dependent DNA replication (GO:0006261), and DNA replication initiation (GO:0006270); those for cellular component (CC) were nucleus (GO:0005634), and chromosome (GO:0005694); those for molecular function (MF) were DNA binding (GO:0003677), DNA binding transcription factor activity (GO:0003700), and transcription regulator activity (GO:0140110). The top enriched GO terms for BP in SSh4 vs. MSh4, were lipid localization (GO:0010876), and lipid transport (GO:0006869); CC were external encapsulating structure (GO:0030312), and cell wall (GO:0005618); those for MF were catalytic activity (GO:0003824), hydrolase activity (GO:0004553, GO:0016798, GO:0016787), and oxidoreductase activity (GO:0016491). The top enriched GO terms for BP in MSh4 vs. MSh2, were DNA replication (GO:0006260), and protein phosphorylation (GO:0006468); those for MF were ATP binding (GO:0005524), and protein kinase activity (GO:0004672). In SSh4 vs. SSh2, the top enriched GO terms for BP were obsolete oxidation reduction process (GO:0055114), lipid localization (GO:0010876), and lipid transport (GO:0006869); those for CC were extracellular region (GO:0005576); those for MF were oxidoreductase activity (GO:0016491, GO:0016701, GO:0016702), and enzyme inhibitor activity (GO:0004857). For SR2 vs. MR2, the top enriched GO terms for BP category were regulation of transcription, DNA templated (GO:0006355), regulation of RNA biosynthetic process (GO:2001141), and regulation of nucleic acid templated transcription (GO:1903506); those for MF were transcription regulator activity (GO:0140110), DNA binding transcription factor activity (GO:0003700), and sequence specific DNA binding (GO:0043565). The top enriched GO terms for BP in SR4 vs. MR4 were response to oxidative stress (GO:0006979), and regulation of transcription, DNA templated (GO:0006355); those for CC were extracellular region (GO:0005576); those for MF were DNA binding transcription factor activity (GO:0003700), and transcription regulator activity (GO:0140110). The top enriched GO terms for BP in MR4 vs. MR2, were response to biotic stimulus (GO:0009607), obsolete oxidation reduction process (GO:0055114); those for CC were apoplast (GO:0048046), extracellular region (GO:0005576); those for MF were nutrient reservoir activity (GO:0045735), and cation binding (GO:0043169). In SR4 vs. SR2, the top enriched GO terms for BP were response to biotic stimulus (GO:0009607), and carbohydrate metabolic process (GO:0005975); those for MF were oxidoreductase activity (GO:0016705, GO:0016491), and cation binding (GO:0043169).

Kyoto encyclopedia of genes and genomes (KEGG) enrichment analysis in rice shoots under NaCl stress is shown in Figure 4C and Table S3. In 9311-2x, DNA replication, DNA repair and recommendation proteins, plant hormone signal transmission, transcription factors, and other pathways were significantly enriched. In 9311-4x, metabolism of terpenoids and polyketides, biosynthesis of other secondary metabolites, transcription factors, cytochrome P450, lipid metabolism, and other pathways were significantly enriched. For the comparison of 9311-4x and 9311-2x, DNA replication, phenylpropanoid biosynthesis and other pathways were significantly enriched in MSh4 vs. MSh2; and biosynthesis of other secondary metals, metabolism of terpenoids and polyketides, phenylpropanoid biosynthesis and other pathways were significantly enriched in SSh4 vs. SSh2. The KEGG enrichment analysis in rice roots under NaCl stress is shown in Figure 4D and Table S3. In SR2 vs. MR2, transcription factors, diterpenoid biosynthesis, phenylpropanoid biosynthesis, signal transduction and other pathways were significantly enriched. In SR4 vs. MR4, phenylpropanoid biosynthesis, biosynthesis of other secondary metabolites, diterpenoid biosynthesis, transcription factors and other pathways were significantly enriched. For the comparison of 9311-4x and 9311-2x, in the mock, diterpenoid biosynthesis, metabolism of terpenoids and polyketides, energy metabolism and other pathways were significantly enriched; in the stress-treated samples, diterpenoid biosynthesis, metabolism of terpenoids and polyketides, cytochrome P450, energy metabolism and other pathways were significantly enriched.

### 2.5. The Differentially Expressed Genes Related to Phytohormones Level in 9311-2x and 9311-4x Rice

**Figure 5:**
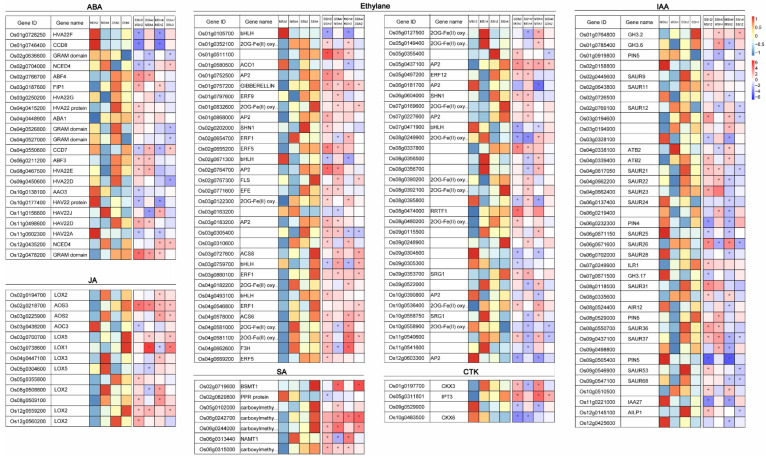
Expression profiles of genes related to phytohormones in shoots between 9311-2x and 9311-4x rice plants under mock and saline stress conditions. Heatmap of differentially expressed genes (DEGs) was generated using MapMan software. Yellow indicates gene expression levels (read_counts per million with log2 value). White indicates the fold change (log2 value) of DEGs. * *p* < 0.05.

To identify the relationship between phytohormone levels and gene expression, we analyzed DEGs related to phytohormone mentalism in shoots (Figure 5, Table S4) and roots (Figure S1, Table S4). In the shoots, the genes involved in ABA metabolism, such as 9-cis-epoxycarotenoid dioxygenase 4 (*NCED4*), abscisic aldehyde oxidase 3 (*AAO3*), carotenoid cleavage dioxygenase 8 (*CCD8*) and carotenoid cleavage dioxygenase 7 (*CCD7*) were all down-regulated, while ABA deficient 1 (*ABA1*) was up-regulated in SSh2 vs. MSh2. Such an observation was not made in SSh4 vs. MSh4. However, the genes involved in ABA signal transduction, such as abscisic acid responsive elements binding factor 3 (*ABF3*) and *ABF4*, were up-regulated in both SSh2 vs. MSh2 and SSh4 vs. MSh4. In addition, a number of ABA-induced genes, such as *GRAM*-domain-containing protein genes, were found to express differentially in SSh2 vs. MSh2 and SSh4 vs. MSh4. *NCED4* and *CCD7* were up-regulated, while numerous *GRAM*-domain-containing protein genes were down-regulated in SSh4 vs. SSh2. Likewise, in the roots, the *NCED3*, *CCD8*, *CCD1*, and *GRAM*-domain-containing protein genes were also up-regulated in SR2 vs. MR2 and SR4 vs. MR4.

ACC synthase 8 (*ACS8*) related to ethylene metabolism was up-regulated in SSh4 vs. MSh4 and SSh4 vs. SSh2 (Figure 5), and *ACS6* was up-regulated in SSh2 vs. MSh2, MSh4 vs. MSh2 and SSh4 vs. SSh2. However, ACC oxidase 1 (*ACO1*) was down-regulated in MSh4 vs. MSh2. *AP2/ERFs* related to ethylene metabolism signal transduction were altered following saline stress in both 9311-2x and 9311-4x, such as *Os01g0797600*, *Os02g0764700*, *Os02g0655200*, *Os02g0764700*, *Os03g0183200*, *Os05g0437100*, and *Os05g0497200*. Basic helix-loop-helix (*bHLH*) involved in ethylene metabolism, including *Os01g0105700*, *Os02g0671300*, *Os03g0759700*, and *Os07g0471900*, were all down-regulated in SSh2 vs. MSh2 and MSh4 vs. MSh2, but there was no significant variation in the comparisons of SSh4 vs. MSh4 and SSh4 vs. SSh2. In roots, most genes were up-regulated in the comparisons of SR2 vs. MR2 and SR4 vs. MR4 (Figure S1).

Indole-3-acetic acid amido synthetase genes, *GH3.2* and *GH3.6*, were up-regulated in Sh4 vs. MSh4, while the former was up-regulated in SSh4 vs. SSh2, and the latter was down-regulated in MSh4 vs. MSh2. Most small auxin up-regulated RNA (*SAUR*) genes were up-regulated in SSh2 vs. MSh2, but down-regulated in SSh4 vs. SSh2. *ILR1*, which is related to auxin metabolism, was up-regulated in SSh2 vs. MSh2. Pin formed 5 (*PIN5*), which is related to auxin signal transduction, was down-regulated in SSh4 vs. SSh2.

Isopentenyl transferase 3 (*IPT3*) was up-regulated in SSh2 vs. MSh2, MSh4 vs. MSh2, and SSh4 vs. SSh2. Both cytokinin oxidase 3 (*CKX3*) and cytokinin oxidase 6 (*CKX6*) were down-regulated in SSh4 vs. MSh4. The genes related to jasmonate synthesis-degradation, such as most of allene oxide synthase (*AOS*) and lipoxygenase (*LOX*), and a number of salicylic acid carboxyl methyltransferase genes were up-regulated in both shoots and roots following saline stress, or in 9311-4x shoots.

## 3. Discussion

WGD has been found to play a crucial role in the development of stress resistance in domesticated plants [45]. Allopolyploids contain multiple sets of chromosomes from two or more distinct yet related species, contributing to plant evolution. However, there is increasing evidence that the evolutionary advantage of autotetraploid plants may have been underestimated [7,8]. In addition, autotetraploid plants have been commonly induced by colchicine treatment, and employed in plant breeding schemes. It is therefore desirable to explore the molecular mechanism that underlie autopolyploidization. Autotetraploid plants were found to be more resilient to environmental stresses than their diploid donor, as exemplified in autotetraploid Arabidopsis, which was found to be more robust than its diploid precursor under salt stress treatment [12]. Autotetraploidization in rice and citrange was also found to be associated with a higher level of resistance to abiotic stresses, which involved alterations in the expression profiles of the genes associated with phytohormone-mediated signaling and metabolic pathways [46,47]. The response of autotetraploid indica rice and its diploid donor to alkaline treatment has been investigated in the genome-wide manner [14]. In japonica rice, polyploidization induces DNA hypomethylation, which co-exists with the expression of stress-responsive genes, such as those involved in the JA pathway, resulting in increased salt tolerance in tetraploid rice compared to diploids [44]. In addition, the difference in stress tolerance between the plants with different ploidy levels was reportedly attributable to the variation in leaf transpiration rate [48,49,50]. Autotetraploid Pak Choi also showed alteration in hormone levels in response to increased drought stress [2,51]. It is conceivable that that more than two alleles per locus as a result of genome doubling renders it possible to produce a variety of allozymes, in addition to the novel functions gained through gene redundance and functional divergence and the reinforcement of the regulatory network in coping with environmental stresses [9].

In this study, after being subjected to short-term saline stress, an eclectic range of plant morphologic traits, including plant height, root length, fresh weight, and dry weight, did not differ significantly between 9311-2x and 9311-4x plants upon 24 h of NaCl treatment. However, the concentration of Na^+^ and K^+^, the content of proline, soluble sugars, MDA, the activity of SOD and POD, as well as the content of numerous phytohormones, including ABA, ACC, IAA, tZ, SA, and JA, were all substantially altered.

Under normal growth conditions, plant height, leaf width, root length, and plant weight were significantly lower in 9311-2x than in 9311-4x at the 3-leaf stage. Following NaCl treatment for 7 d, 9311-2x plants showed exacerbated wilting and decreased fresh and dry weights. In contrast, 9311-4x plants exhibited fewer stomata per unit area, resulting in decreased transpiration rate in response to salt and drought stress [52], suggesting a potential cause of the greater water loss in 9311-2x. This is also consistent with the observation in japonica rice that tetraploid plants are more drought resistant than their diploid counterparts [53]. In the present study, with 9311-2x and 9311-4x, there were also rapid alterations in the content of proline, soluble sugars, and MDA in the first 24 h after NaCl treatment, but without a consistent pattern. However, the activities of SOD and POD were consistently increasing in 9311-4x following NaCl treatment for 6 h. In response to salt stress, SOD converts elevated superoxide molecules into oxygen and H_2_O_2_, whereas POD converts H_2_O_2_ into oxygen and water [18]. It has been widely reported that SOD and POD scavenge the ROS in plant cells to mitigate abiotic stress [21,54,55]. The observed elevation in the activities of SOD and POD might be attributable to the enhancement in salt tolerance in 9311-4x. As is evident in Figure S2 and Table S5, numerous peroxidase genes were up-regulated in SSh4 vs. SSh2 (*Os04g0688100*, *Os05g0134700*, *Os12g0111800*, etc.) and in SSh4 vs. MSh4 (*Os02g0240100*, *Os04g0651000*, *Os12g01118000*, etc.), whereas others were down-regulated in SSh2 vs. MSh2 (*Os02g0161800*, *Os03g0563600*, *Os07g0677200*, etc.) and in MSh4 vs. MSh2 (*Os01g0327000*, *Os07g0677200*, *Os10g0109600*, etc.), supporting the notion that an elevated oxidase activity in 9311-4x may have conferred higher tolerance to salt stress relative to 9311-2x.

While the concentration of Na^+^ and K^+^ rose rapidly in response to saline stress, 9311-4x exhibited a greater capacity to maintain ionic equilibrium than its diploid counterparts, corroborating a previous report from the wheat literature that the increase in K^+^ exceeded that of Na^+^ [56]. It was reported that Arabidopsis polyploids with improved saline tolerance benefited from increased K^+^ uptake [12]. The K^+^ concentration in 9311-4x increased rapidly in the first 6 h upon NaCl treatment, but then leveled off. Maintaining a high ratio of cytosolic K^+^/Na^+^ ratio is essential for plant development. Salt stress would disrupt the steady flow of Na^+^ into the cytoplasm and activate K^+^ outflow channels [18]. Our data demonstrated that the K^+^/Na^+^ ratio decreased rapidly after exposure to NaCl treatment, and that a significant increase in 9311-4x relative to 9311-2x occurred between 6 h and 24 h following treatment, sending a clear message that the former was more salt tolerant than the latter. The expression of genes involved in high-affinity K^+^ transport, including those encoding high-affinity K^+^ transporters (HKTs) and high-affinity K^+^ absorption transport channel proteins (HAKs) was altered by saline stress (Figure S3, Table S5). In the meantime, glutamine synthetase (GS) and glutamate dehydrogenase (GDH) in the nitrogen metabolism pathway converted NH^4+^ to amino acid in response to saline stress [57,58]. In this study, *OsHAK9* (*Os07g0679000*) exhibited a significant increase in SSh4 vs. SSh2, indicating its potential contribution to a higher concentration of K^+^ in the shoots of 9311-4x relative to 9311-2x upon NaCl treatment for 6 h. *OsGS1;3* (*Os03g0712800*) was reported to be spikelet specific [59], but saline stress strongly induced its expression in the roots of the diploid and the shoots of the autotetraploid rice. *OsGDH3* (*Os02g0650900*), which was reported to be expressed in the senescent leaves of rice [57], was also induced by saline stress to be expressed in the shoots of autotetraploid rice. Both *OsGS1;3* and *OsGDH3* were markedly up-regulated in SSh4 vs. SSh2. *OsNHX1* (*Os07g0666900*), which encodes Na^+^/H^+^ exchanger [60], was expressed > 2 folds higher in SSh4 vs. MSh4, potentially contributing to salt tolerance in the autotetraploid rice.

GO and KEGG enrichment analyses on DEGs that were displayed in the transcriptomes shed more light on the divergent responses of 9311-4x and 9311-2x to saline stress. The GO results showed that genes in the pathways of cyclic compounds, ion binding and oxidoreduction were significantly enriched under salt stress. Further, amine-related genes were significantly enriched in roots, while organic acid and lipid metabolism related genes were significantly enriched in the shoots in both 9311-4x and 9311-2x, with the former significantly higher than the latter. The KEGG analysis showed that terpenoids, transcription factors, and hormone and MAPK signaling pathways were all significantly up-regulated, suggestive of their roles in salt resistance in rice. Significantly enriched in tetraploid shoots were genes involved in lipid metabolism, cutin, suberine and wax biosynthesis, cytochrome P450, and phenylpropanoid-related pathways. The primary component of cell membranes and the alteration in lipid composition caused by salt stress can affect membrane fluidity and the functionality of membrane-bound enzymes. In response to saline stress, plants may alter the expression of many genes involved in lipid metabolism to maintain membrane hemostasis, in addition to initiating the signal cascade reaction [61]. Moreover, organic acids, cytochrome P450 [62] and phenylpropanoid [63] can also participate in plants’ responses to various abiotic stresses; however, the similarity and specificity of these genes and pathways in rice, relative to other plants or other stress factors, remain to be further explored.

The genes involved in phytohormone metabolism and accumulation were differentially expressed in the shoots and roots between 9311-4x and 9311-2x. *ABFs* involved in ABA transduction and regulation of stomatal closure exhibited significantly increased expression in SSh4 and SSh2, corroborating a previous study that demonstrated their positive response to osmotic stress in plants [64]. Likewise, *ABA1* was highly expressed in both SSh4 and SSh2, and differentially expressed in SSh2 vs. MSh2. *NCED4*, an additional key gene involved in ABA biosynthesis, was significantly up-regulated in SSh4 vs. SSh2, in line with a previous report that *NCED3* was up-regulated by water deficiency [65]. *CCD7* and *CCD8* were two strigolactone (*SL*) biosynthesis genes, which exhibited greater expression in 9311-4x than in 9311-2x following saline stress, validating *SL*’s role in mediating salt tolerance in an ABA-dependent manner.

Both *ACS6* and *ACS8* were significantly up-regulated in SSh4 vs. SSh2, which can be attributed to the higher ACC level in 9311-4x as result of NaCl treatment. *AP2/ERF* transcription factors that are involved in ethylene transduction displayed significant variation in response to NaCl treatment in both 9311-4x and 9311-2x plants. Moreover, the expression of an additional ethylene responsive gene, *bHLH*, was altered by saline stress, indicating that the ethylene transduction pathway is involved in regulating saline stress tolerance in rice [18]. In Arabidopsis, the expression of *SAUR* was down-regulated at 72 h after dehydration in WT plants [65]. In the present study, *SAUR*s were up-regulated in both 9311-2x and 9311-4x as a result of NaCl treatment but down-regulated in SSh4 vs. SSh2, which might be attributed to the greater extent of reduction in IAA level in 9311-4x relative to 9311-2x following NaCl treatment for 6 h.

SA catabolism-related genes were significantly up-regulated in SSh4 vs. SSh2 in response to NaCl treatment, which may have contributed to SA level variation. According to a previous study, SA activates the expression of *P5CS*, which is responsible for proline accumulation under saline stress [66,67], corroborating our finding that SA levels were increased proportionally to proline content. In the present study, *OsP5CS* was down-regulated in MSh4 vs. MSh2, but recovered in SSh4 vs. SSh2, which may be due to the relatively higher salt tolerance in 9311-4x compared to 9311-2x.

## 4. Materials and Methods

### 4.1. NaCl Treatment

9311-4x is a typical autotetraploid rice arose from chromosome-doubling of its donor 9311-2x as previously described [14]. The seeds of both 9311-4x and 9311-2x were germinated for 2 d on moist tissue towels at 28 °C in the dark. The seedlings were transferred to a half-strength Hoagland nutrient solution for further growth, with continuous fluorescent lighting under a temperature cycle of 28/26 °C for 16/8 h and relative humidity maintained at approximately 70% as described in our previous report [14]. When the seedlings reached 3-leaf stage, the growth medium of half number of the plants was supplemented with NaCl to the final concentration of 100 mM for NaCl treatment for 1 d or 7 d. The remaining plants were kept in the original culture solution under the mock growth conditions as a control. At the end of NaCl treatment, both NaCl-treated and mock rice seedlings of 9311-2x and 9311-4x were harvested and a range of phenotypic traits, including plant height, root length, fresh weight, and dry weight, were measured as previously described [14]. Three biological replicates were conducted, each of which contained three technical replicates.

### 4.2. The Measurements of Ionic Concentration, Small-Molecule Organic Compounds and Enzyme Activity

To measure ionic concentration, shoots of 9311-2x and 9311-4x seedlings were sampled following NaCl treatment for 0 h, 3 h, 6 h, 12 h and 24 h and dried at 75 °C for 2 d in a baking oven. Approximately 0.1 g dried plant tissues were then ground to powder before being mixed with 6 mL of nitric acid and 2 mL of perhydrol (Sigma Aldrich, St. Louis, MO, USA) in a microwave at 100 °C for digestion. Total ion content was determined using inductively coupled plasma mass spectrometry (ICAP6300; Thermo Scientific, Waltham, MA, USA) as previously described [54].

The content of proline, soluble sugars, MDA, and the enzyme activities of SOD and POD were analyzed using a SmartSpecTM Plus spectrophotometer (BioRad, Hercules, CA, USA) [68,69,70]. Proline was determined based on the test method described by Bates et al. [70]. Approximately 5 mL of 3% sulfosalicylic acid was added to a 0.5 g fresh leaf sample and vigorously mixed for 10 min before being filtered. The proline content in the filtrate was then measured at a wavelength of 520 nm using the standard curve using a spectrophotometer. To measure the content of the soluble sugars in plant leaf, 0.5 mL of anthrone reagent and 5 mL of concentrated H_2_SO_4_ were mixed with leaf tissues and incubated in a boiling water bath for 10 min. Absorbance at 625 nm was measured with a spectrophotometer and the sugar content was determined by comparison to the standard curve as previously described [71]. For the determination of MDA content, a 0.5 g leaf sample was homogenized with 10% trichloroacetic acid before being centrifuged at 4000× rpm for 10 min. To the supernatant, a 0.6% thiobarbituric acid solution was added, and allowed to react in a boiling water bath for 15 min. The absorbance was measured at 532 nm, 600 nm, and 450 nm [72]. SOD activity was measured based on NBT photochemical reduction, and POD activity was measured based on absorbance induced by guaiacol, as previously described [73,74]. Briefly, 0.2 g leaf tissues were homogenized in 50 mL of 1% polyvinylpyrrolidone in phosphate buffer (pH = 7.8), before being centrifuged at 12,000× *g* for 20 min at 4 °C. The absorbance was then measured at 560 nm and 470 nm and the enzyme activities were calculated as previously described [73,74]. All the analyses were conducted with three independent biological replicates, each of which had three technical replicates.

### 4.3. Phytohormones Assay

Phytohormones were analyzed using an ACQUITY UHPLC system (Waters Corporation, Milford, MA, USA) coupled with an AB SCIEX API 5500 System (AB SCIEX, Framingham, MA, USA), and the profile analysis was conducted using the Analyst 1.6.2 workstation (AB SCIEX). All assays consisted of three biological replicates, each of which contained three technical replicates.

### 4.4. RNA Isolation and RNA Sequencing

Shoots and roots of 9311-2x and 9311-4x seedlings were harvested immediately after the designated NaCl treatment for 0, 3, 6, 12 and 24 h. Total RNA was isolated with the Trizol reagent (Invitrogen, Carlsbad, CA, USA) following the manufacturer’s instruction. The RNA was then treated with RNase-free DNaseI (Invitrogen) to eliminate potential genomic DNA contamination before being reverse-transcribed with the Super Script RNase H-Reverse Transcriptase (Invitrogen). The cDNA library construction was carried out following the Illumina sample preparation protocol (Illumina, San Diego, CA, USA), and the samples were sequenced using the Novaseq platform. Clean reads were generated by removing adaptor sequences, low-quality sequences, and polyA sequences. BWA and STAR were used to map clean reads to the reference genome [75] as previously described [14]. The DESeq2 R package was used to normalize the data and calculate differentially expressed genes with max_readcount > 30, foldchange > 2, and *p* < 0.05. The experiment contained three biological replicates. Clean data has been deposited in the SRA database with accession numbers, PRJNA812638 and PRJNA856424 (http://www.ncbi.nlm.nih.gov/sra/, accessed on 5 May 2022 and 8 July 2022, respectively).

### 4.5. GO Classification and KEGG Classification Analysis

Molecular function category GO terms were analyzed by using the online PANTHER 15.0 platform (http://www.pantherdb.org/, accessed on 20 March 2022) with an FDR-corrected *p* value < 0.05 (Fisher tests). Pathway assignments were performed following KEGG mapping (http://www.genome.ad.jp/kegg/kegg2.html, accessed on 20 March 2022) as previously described [76].

### 4.6. qRT-PCR Validation

qRT-PCR was used to validate the RNA-seq data. Ten DEGs were randomly selected and analyzed as previously described [14]. The SYBR Green I PCR master mix kit (TaKaRa, Tokyo, Japan) was used in the qRT-PCR reactions [14]. The experiment contained three biological replicates. Gene-specific primer pairs were designed following our previous report [14]. The results of qRT-PCR were listed in Figure S4.

### 4.7. Statistical Analysis

All statistical analyses were conducted using t-tests and the results were expressed as the mean ± standard deviation of three biological replicates. The statistical significance threshold was set at *p* value < 0.05.

## 5. Conclusions

Plant polyploidization confers an apparent evolutionary advantage, expanding genetic reservoir and bolstering combinatorial complexity, which is a necessity for plants to thrive and develop under an increasingly harsh environment [7,77]. It is therefore highly desirable to explore the advantage of polyploidization and its underlying mechanisms [78]. In this study, we investigated and compared plant morphology, physiology parameters, phytohormones levels, and gene expression variations in the shoot and root tissues of autotetraploid indica rice and its diploid donor. It is apparent that the autotetraploid rice was more resistant to short-term exposure to saline stress than its diploid donor. Rice plants underwent a rapid elevation in the accumulation of small molecule organic compounds, Na^+^, K^+^ and phytohormones in response to saline stress. The expression of the genes that are associated with oxidase activity and phytohormones was substantially higher in the autotetraploid than the diploid rice, which is of particular interest. It is understood that the high-throughput methods used in this study may not always be directly associated with the metabolic fluxes in response to environmental cues in a quantitative manner. Such a limitation mandate cautious interpretations of the findings in the current study. Therefore, it would be more judicious to explore the detailed kinetic and regulatory networks under saline stress in the future. Nevertheless, our findings in this study may shed additional light on the mechanism underlying plant tolerance to saline stress, particularly in relation to polyploidization, and suggest a potentially fruitful avenue for further exploration.

## Data Availability

The datasets generated and analyzed in this study are available at [PRJNA856424 and PRJNA812638] https://www.ncbi.nlm.nih.gov/sra/PRJNA856424 (access on 5 May 2022) and https://www.ncbi.nlm.nih.gov/sra/PRJNA812638 (accessed on 8 July 2022).

## Supplementary Materials

The following supporting information can be downloaded at: https://www.mdpi.com/article/10.3390/plants11223114/s1, Figure S1: Expression profiles of genes related to phytohormones in shoots of 9311-2x and 9311-4x rice plants under mock and saline stress conditions; Figure S2: Expression pro-files of genes related to peroxidase superfamily in shoots and roots of 9311-2x and 9311-4x rice plants under mock and saline stress conditions; Figure S3: Expression pro-files of genes related to ionic transport in shoots and roots of 9311-2x and 9311-4x rice plants under mock and saline stress conditions; Figure S4: The expression of ten randomly selected genes was analyzed using quantitative reverse-transcriptase polymerase chain reaction (qRT-PCR) for the verification of the transcriptome results; Table S1: RNA-seq data quality control; Table S2: The list of DEGs in GO terms; Table S3: The list of DEGs in KEGG terms; Table S4: The list of DEGs with phytohormone-related genes; Table S5: The list of DEGs with peroxidase superfamily protein and ionic transport-related genes, etc.

## References

[1] Wu Y., Lin F., Zhou Y., Wang J., Sun S., Wang B., Zhang Z., Li G., Lin X., Wang X. (2021). Genomic mosaicism due to homoeologous exchange generates extensive phenotypic diversity in nascent allopolyploids. Natl. Sci. Rev..

[2] Zhang C., Wang H., Xu Y., Zhang S., Wang J., Hu B., Hou X., Li Y., Liu T. (2020). Enhanced Relative Electron Transport Rate Contributes to Increased Photosynthetic Capacity in Autotetraploid Pak Choi. Plant Cell Physiol..

[3] Fawcett J.A., Maere S., Van de Peer Y. (2009). Plants with double genomes might have had a better chance to survive the Cretaceous-Tertiary extinction event. Proc. Proc. Natl. Acad. Sci. USA.

[4] Xiao L., Lu L., Zeng W., Shang X., Cao S., Yan H. (2022). DNA Methylome and LncRNAome Analysis Provide Insights into Mechanisms of Genome-Dosage Effects in Autotetraploid Cassava. Front. Plant Sci..

[5] Braynen J., Yang Y., Yuan J., Xie Z., Cao G., Wei X., Shi G., Zhang X., Wei F., Tian B. (2021). Comparative transcriptome analysis revealed differential gene expression in multiple signaling pathways at flowering in polyploid Brassica rapa. Cell Biosci..

[6] Haldane J.B.S. (1933). The part played by recurrent mutation in evolution. Am. Nat..

[7] Zhang J., Liu Y., Xia E.H., Yao Q.Y., Liu X.D., Gao L.Z. (2015). Autotetraploid rice methylome analysis reveals methylation variation of transposable elements and their effects on gene expression. Proc. Natl. Acad. Sci. USA.

[8] Parisod C., Holderegger R., Brochmann C. (2010). Evolutionary consequences of autopolyploidy. New Phytol..

[9] Liu B., Sun G. (2017). microRNAs contribute to enhanced salt adaptation of the autopolyploid *Hordeum bulbosum* compared with its diploid ancestor. Plant J..

[10] Tossi V.E., Martinez Tosar L.J., Laino L.E., Iannicelli J., Regalado J.J., Escandon A.S., Baroli I., Causin H.F., Pitta-Alvarez S.I. (2022). Impact of polyploidy on plant tolerance to abiotic and biotic stresses. Front. Plant Sci..

[11] Da L. (1983). Polyploidy and novelty in flowering plants. Am. Nat..

[12] Chao D.Y., Dilkes B., Luo H.B., Douglas A., Yakubova E., Lahner B., Salt D.E. (2013). Polyploids Exhibit Higher Potassium Uptake and Salinity Tolerance in Arabidopsis. Science.

[13] Nassar N.M., Graciano-Ribeiro D., Fernandes S.D.C., Araujo P.C. (2008). Anatomical alterations due to polyploidy in cassava, Manihot esculenta Crantz. Genet. Mol. Res..

[14] Wang N., Fan X., Lin Y., Li Z., Wang Y., Zhou Y., Meng W., Peng Z., Zhang C., Ma J. (2022). Alkaline Stress Induces Different Physiological, Hormonal and Gene Expression Responses in Diploid and Autotetraploid Rice. Int. J. Mol. Sci..

[15] Wang Z.M., Wang M.Y., Liu L., Meng F.J. (2013). Physiological and Proteomic Responses of Diploid and Tetraploid Black Locust (*Robinia pseudoacacia* L.) Subjected to Salt Stress. Int. J. Mol. Sci..

[16] del Pozo J.C., Ramirez-Parra E. (2014). Deciphering the molecular bases for drought tolerance in Arabidopsis autotetraploids. Plant Cell Environ..

[17] Shabala S. (2013). Learning from halophytes: Physiological basis and strategies to improve abiotic stress tolerance in crops. Ann. Bot..

[18] Fang S., Hou X., Liang X. (2021). Response Mechanisms of Plants Under Saline-Alkali Stress. Front. Plant Sci..

[19] Oster J., Shainberg I., Abrol I. (1999). Reclamation of salt-affected soils. Agric. Drain..

[20] Qiu Q.S., Guo Y., Quintero F.J., Pardo J.M., Schumaker K.S., Zhu J.K. (2004). Regulation of vacuolar Na+/H+ exchange in Arabidopsis thaliana by the salt-overly-sensitive (SOS) pathway. J. Biol. Chem..

[21] An M., Wang X., Chang D., Wang S., Hong D., Fan H., Wang K. (2020). Application of compound material alleviates saline and alkaline stress in cotton leaves through regulation of the transcriptome. BMC Plant Biol..

[22] Munns R., Tester M. (2008). Mechanisms of salinity tolerance. Annu. Rev. Plant Biol..

[23] Wang R., Jing W., Xiao L., Jin Y., Shen L., Zhang W. (2015). The Rice High-Affinity Potassium Transporter1;1 Is Involved in Salt Tolerance and Regulated by an MYB-Type Transcription Factor. Plant Physiol..

[24] Tester M., Davenport R. (2003). Na^+^ tolerance and Na^+^ transport in higher plants. Ann. Bot..

[25] Deinlein U., Stephan A.B., Horie T., Luo W., Xu G., Schroeder J.I. (2014). Plant salt-tolerance mechanisms. Trends Plant Sci..

[26] Sun J., He L., Li T. (2019). Response of seedling growth and physiology of *Sorghum bicolor* (L.) Moench to saline-alkali stress. PLoS ONE.

[27] Hazman M., Hause B., Eiche E., Nick P., Riemann M. (2015). Increased tolerance to salt stress in OPDA-deficient rice ALLENE OXIDE CYCLASE mutants is linked to an increased ROS-scavenging activity. J. Exp. Bot..

[28] Szymanska K.P., Polkowska-Kowalczyk L., Lichocka M., Maszkowska J., Dobrowolska G. (2019). SNF1-Related Protein Kinases SnRK2.4 and SnRK2.10 Modulate ROS Homeostasis in Plant Response to Salt Stress. Int. J. Mol. Sci..

[29] Niu M., Xie J., Chen C., Cao H., Sun J., Kong Q., Shabala S., Shabala L., Huang Y., Bie Z. (2018). An early ABA-induced stomatal closure, Na+ sequestration in leaf vein and K+ retention in mesophyll confer salt tissue tolerance in Cucurbita species. J. Exp. Bot..

[30] Chen K., Li G.J., Bressan R.A., Song C.P., Zhu J.K., Zhao Y. (2020). Abscisic acid dynamics, signaling, and functions in plants. J. Integr. Plant Biol..

[31] Yang S., Yu Q., Zhang Y., Jia Y., Wan S., Kong X., Ding Z. (2018). ROS: The Fine-Tuner of Plant Stem Cell Fate. Trends Plant Sci..

[32] Han J.P., Koster P., Drerup M.M., Scholz M., Li S., Edel K.H., Hashimoto K., Kuchitsu K., Hippler M., Kudla J. (2019). Fine-tuning of RBOHF activity is achieved by differential phosphorylation and Ca^2+^ binding. New Phytol..

[33] Peng J., Li Z., Wen X., Li W., Shi H., Yang L., Zhu H., Guo H. (2014). Salt-induced stabilization of EIN3/EIL1 confers salinity tolerance by deterring ROS accumulation in Arabidopsis. PLoS Genet..

[34] Zhang M., Smith J.A., Harberd N.P., Jiang C. (2016). The regulatory roles of ethylene and reactive oxygen species (ROS) in plant salt stress responses. Plant Mol. Biol..

[35] Liu W., Li R.J., Han T.T., Cai W., Fu Z.W., Lu Y.T. (2015). Salt stress reduces root meristem size by nitric oxide-mediated modulation of auxin accumulation and signaling in Arabidopsis. Plant Physiol..

[36] Lv B., Yan Z., Tian H., Zhang X., Ding Z. (2019). Local Auxin Biosynthesis Mediates Plant Growth and Development. Trends Plant Sci..

[37] Nishiyama R., Watanabe Y., Fujita Y., Le D.T., Kojima M., Werner T., Vankova R., Yamaguchi-Shinozaki K., Shinozaki K., Kakimoto T. (2011). Analysis of cytokinin mutants and regulation of cytokinin metabolic genes reveals important regulatory roles of cytokinins in drought, salt and abscisic acid responses, and abscisic acid biosynthesis. Plant Cell.

[38] Hyoung S., Cho S.H., Chung J.H., So W.M., Cui M.H., Shin J.S. (2020). Cytokinin oxidase PpCKX1 plays regulatory roles in development and enhances dehydration and salt tolerance in *Physcomitrella patens*. Plant Cell Rep..

[39] Abouelsaad I., Renault S. (2018). Enhanced oxidative stress in the jasmonic acid-deficient tomato mutant def-1 exposed to NaCl stress. J. Plant Physiol..

[40] Kurotani K., Hayashi K., Hatanaka S., Toda Y., Ogawa D., Ichikawa H., Ishimaru Y., Tashita R., Suzuki T., Ueda M. (2015). Elevated levels of CYP94 family gene expression alleviate the jasmonate response and enhance salt tolerance in rice. Plant Cell Physiol..

[41] Ahanger M.A., Aziz U., Alsahli A.A., Alyemeni M.N., Ahmad P. (2019). Influence of Exogenous Salicylic Acid and Nitric Oxide on Growth, Photosynthesis, and Ascorbate-Glutathione Cycle in Salt Stressed Vigna angularis. Biomolecules.

[42] Yu Z., Duan X., Luo L., Dai S., Ding Z., Xia G. (2020). How Plant Hormones Mediate Salt Stress Responses. Trends Plant Sci..

[43] Kaur H., Sirhindi G., Bhardwaj R., Alyemeni M.N., Siddique K.H.M., Ahmad P. (2018). 28-homobrassinolide regulates antioxidant enzyme activities and gene expression in response to salt- and temperature-induced oxidative stress in Brassica juncea. Sci. Rep..

[44] Wang L., Cao S., Wang P., Lu K., Song Q., Zhao F.J., Chen Z.J. (2021). DNA hypomethylation in tetraploid rice potentiates stress-responsive gene expression for salt tolerance. Proc. Natl. Acad. Sci. USA.

[45] Renny-Byfield S., Wendel J.F. (2014). Doubling down on genomes: Polyploidy and crop plants. Am. J. Bot..

[46] Yang P.M., Huang Q.C., Qin G.Y., Zhao S.P., Zhou J.G. (2014). Different drought-stress responses in photosynthesis and reactive oxygen metabolism between autotetraploid and diploid rice. Photosynthetica.

[47] Ruiz M., Quinones A., Martinez-Cuenca M.R., Aleza P., Morillon R., Navarro L., Primo-Millo E., Martinez-Alcantara B. (2016). Tetraploidy enhances the ability to exclude chloride from leaves in carrizo citrange seedlings. J. Plant Physiol..

[48] Maherali H., Walden A.E., Husband B.C. (2009). Genome duplication and the evolution of physiological responses to water stress. New Phytol..

[49] Deng B.L., Du W.C., Liu C.L., Sun W.W., Tian S., Dong H.S. (2012). Antioxidant response to drought, cold and nutrient stress in two ploidy levels of tobacco plants: Low resource requirement confers polytolerance in polyploids?. Plant Growth Regul..

[50] Soltis P.S., Soltis D.E. (2014). Flower diversity and angiosperm diversification. Methods Mol. Biol..

[51] Wu H., Song X., Lyu S., Ren Y., Liu T., Hou X., Li Y., Zhang C. (2022). Integrated Analysis of Hi-C and RNA-Seq Reveals the Molecular Mechanism of Autopolyploid Growth Advantages in Pak Choi (*Brassica rapa* ssp. chinensis). Front. Plant Sci..

[52] Li W.L., Berlyn G.P., Ashton P.M.S. (1996). Polyploids and their structural and physiological characteristics relative to water deficit in *Betula papyrifera* (Betulaceae). Am. J. Bot..

[53] Li W.-D., Biswas D.K., Xu H., Xu C.-Q., Wang X.-Z., Liu J.-K., Jiang G.-M. (2009). Photosynthetic responses to chromosome doubling in relation to leaf anatomy in Lonicera japonica subjected to water stress. Funct. Plant Biol..

[54] Li Q., Yang A., Zhang W.H. (2017). Comparative studies on tolerance of rice genotypes differing in their tolerance to moderate salt stress. BMC Plant Biol..

[55] Liu D., Liu M., Liu X.L., Cheng X.G., Liang Z.W. (2018). Silicon Priming Created an Enhanced Tolerance in Alfalfa (*Medicago sativa* L.) Seedlings in Response to High Alkaline Stress. Front. Plant Sci..

[56] Yang C., Zhao L., Zhang H., Yang Z., Wang H., Wen S., Zhang C., Rustgi S., von Wettstein D., Liu B. (2014). Evolution of physiological responses to salt stress in hexaploid wheat. Proc. Natl. Acad. Sci. USA.

[57] Wang H., Zhang M., Guo R., Shi D.C., Liu B., Lin X., Yang C. (2012). Effects of salt stress on ion balance and nitrogen metabolism of old and young leaves in rice (Oryza sativa L.). BMC Plant Biol..

[58] Wang H., Wu Z., Han J., Zheng W., Yang C. (2012). Comparison of ion balance and nitrogen metabolism in old and young leaves of alkali-stressed rice plants. PLoS ONE.

[59] Kusano M., Tabuchi M., Fukushima A., Funayama K., Diaz C., Kobayashi M., Hayashi N., Tsuchiya Y.N., Takahashi H., Kamata A. (2011). Metabolomics data reveal a crucial role of cytosolic glutamine synthetase 1;1 in coordinating metabolic balance in rice. Plant J..

[60] Parida A.K., Das A.B. (2005). Salt tolerance and salinity effects on plants: A review. Ecotoxicol. Environ. Saf..

[61] Hou Q., Ufer G., Bartels D. (2016). Lipid signalling in plant responses to abiotic stress. Plant Cell Environ..

[62] Pandian B.A., Sathishraj R., Djanaguiraman M., Prasad P.V.V., Jugulam M. (2020). Role of Cytochrome P450 Enzymes in Plant Stress Response. Antioxidants.

[63] Sharma A., Shahzad B., Rehman A., Bhardwaj R., Landi M., Zheng B. (2019). Response of Phenylpropanoid Pathway and the Role of Polyphenols in Plants under Abiotic Stress. Molecules.

[64] Cai S., Chen G., Wang Y., Huang Y., Marchant D.B., Wang Y., Yang Q., Dai F., Hills A., Franks P.J. (2017). Evolutionary Conservation of ABA Signaling for Stomatal Closure. Plant Physiol..

[65] Urano K., Maruyama K., Jikumaru Y., Kamiya Y., Yamaguchi-Shinozaki K., Shinozaki K. (2017). Analysis of plant hormone profiles in response to moderate dehydration stress. Plant J..

[66] Lee S., Kim S.G., Park C.M. (2010). Salicylic acid promotes seed germination under high salinity by modulating antioxidant activity in Arabidopsis. New Phytol..

[67] Zheng J., Ma X., Zhang X., Hu Q., Qian R. (2018). Salicylic acid promotes plant growth and salt-related gene expression in *Dianthus superbus* L. (Caryophyllaceae) grown under different salt stress conditions. Physiol. Mol. Biol. Plants.

[68] Bates L.S., Waldren R.P., Teare I.D. (1973). Rapid determination of free proline for water-stress studies. Plant Soil.

[69] Song S.Y., Chen Y., Chen J., Dai X.Y., Zhang W.H. (2011). Physiological mechanisms underlying OsNAC5-dependent tolerance of rice plants to abiotic stress. Planta.

[70] Yang A., Dai X., Zhang W.H. (2012). A R2R3-type MYB gene, OsMYB2, is involved in salt, cold, and dehydration tolerance in rice. J. Exp. Bot..

[71] Irigoyen J.J., Emerich D.W., Sanchezdiaz M. (1992). Water stress induced changes in concentrations of proline and total soluble sugars in nodulated alfalfa (*Medicago sativa*) plants. Physiol. Plant..

[72] Heath R.L., Packer L. (1968). Photoperoxidation in isolated chloroplasts: I. kinetics and stoichiometry of fatty acid peroxidation. Arch Biochem. Biophys..

[73] Paoletti F., Aldinucci D., Mocali A., Caparrini A. (1986). A sensitive spectrophotometric method for the determination of superoxide dismutase activity in tissue extracts. Anal. Biochem..

[74] Zhou W., Leul M. (1999). Uniconazole-induced tolerance of rape plants to heat stress in relation to changes in hormonal levels, enzyme activities and lipid peroxidation. Plant Growth Regul..

[75] Zhao Q., Feng Q., Lu H., Li Y., Wang A., Tian Q., Zhan Q., Lu Y., Zhang L., Huang T. (2018). Pan-genome analysis highlights the extent of genomic variation in cultivated and wild rice. Nat. Genet..

[76] Lin Y., Ma J., Wu N., Qi F., Peng Z., Nie D., Yao R., Qi X., Slaski J., Yang F. (2022). Transcriptome Study of Rice Roots Status under High Alkaline Stress at Seedling Stage. Agronomy.

[77] Soltis D.E., Albert V.A., Leebens-Mack J., Bell C.D., Paterson A.H., Zheng C., Sankoff D., Depamphilis C.W., Wall P.K., Soltis P.S. (2009). Polyploidy and angiosperm diversification. Am. J. Bot..

[78] Van de Peer Y., Ashman T.L., Soltis P.S., Soltis D.E. (2021). Polyploidy: An evolutionary and ecological force in stressful times. Plant Cell.

