# Supplementary material for: Autotetraploidization Gives Rise to Differential Gene Expression in Response to Saline Stress in Rice
Source: Plants (Basel). 2022 Nov 15;11(22):3114. doi: 10.3390/plants11223114 (PMC9698567; doi:10.3390/plants11223114)
Supplement: Supplementary file 1 [file plants-11-03114-s001.zip › Figures S1--S4.pdf]

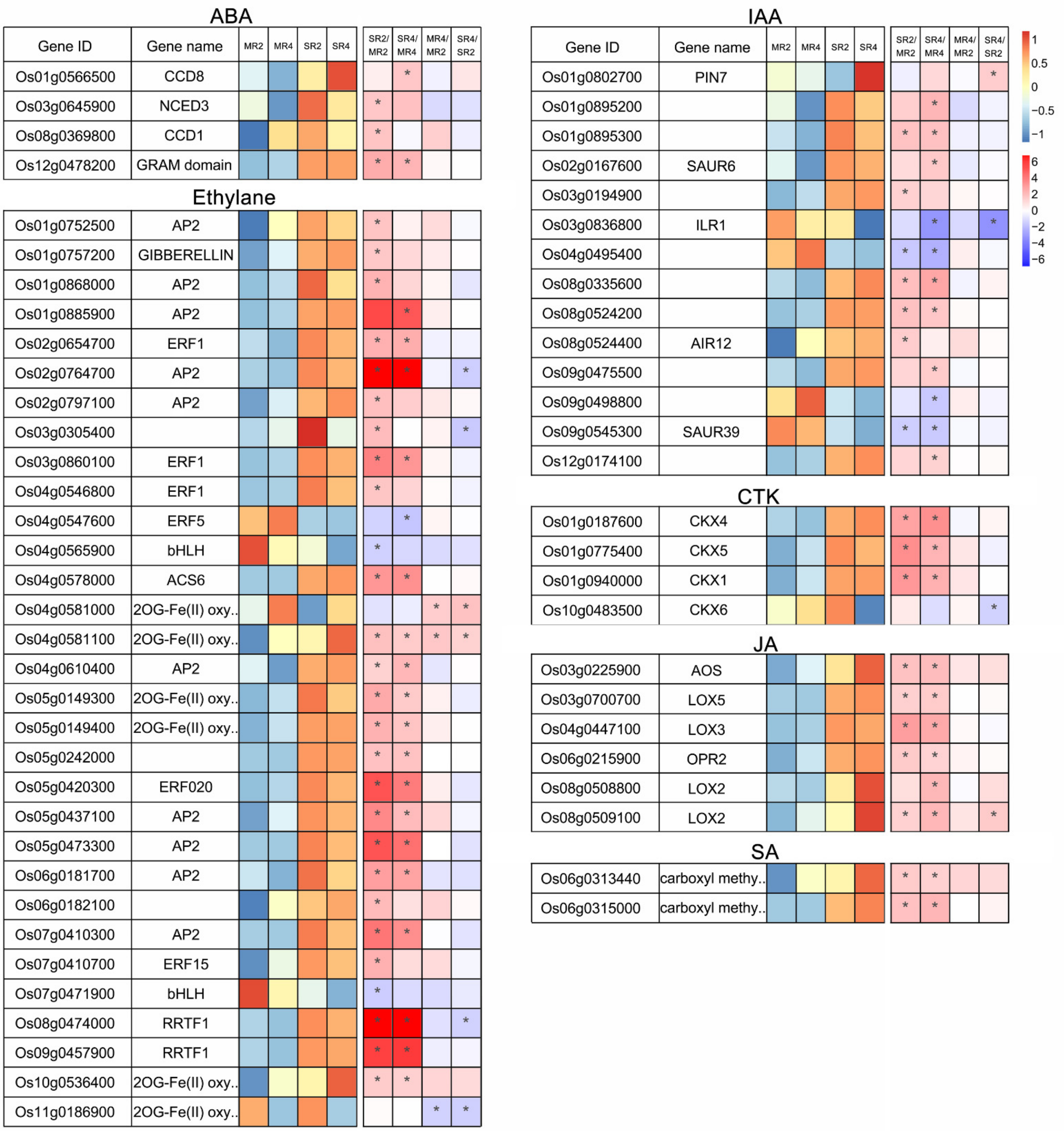

Figure S1. Expression profiles of genes related to phytohormones in shoots of 9311-2x and 9311-4x rice plants under mock and saline stress conditions. Heatmap of differentially expressed genes (DEGs) was generated using MapMan software. Yellow indicates genes expression levels (read_counts per million with log2 value). White indicates the fold change (log2 value) of DEGs. ** $p < 0.01$, * $p < 0.05$. MR2 and MR4 represent the root samples derived from 9311-2x and 9311-4x plants, respectively. SR2 and SR4 represent the saline stress treated root samples derived from 9311-2x and 9311-4x plants, respectively. MSh2 and MSh4 represent the shoot samples derived from 9311-2x and 9311-4x plants, respectively. SSh2 and SSh4 represent the saline stress treated shoot samples derived from 9311-2x and 9311-4x plants, respectively.

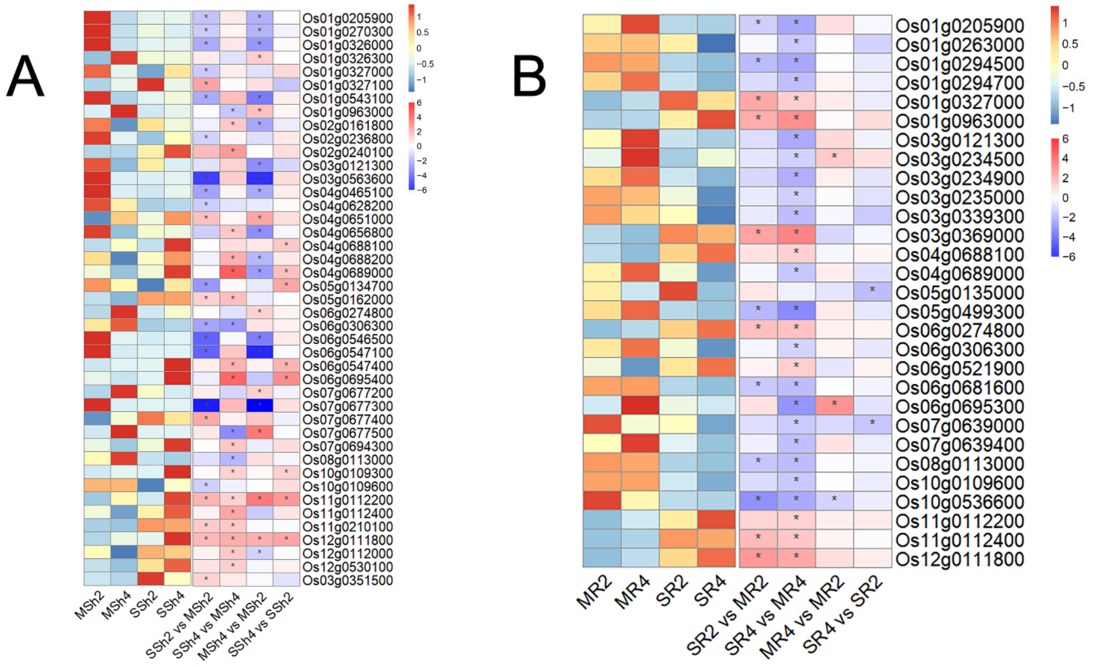

Figure S2. Expression profiles of genes related to peroxidase superfamily in shoots and roots of 9311-2x and 9311-4x rice plants under mock and saline stress conditions. Heatmap of differentially expressed genes (DEGs) was generated using MapMan software. Yellow indicates genes expression levels (read_counts per million with log2 value). White indicates the fold change (log2 value) of DEGs. ** $p < 0.01$, * $p < 0.05$.

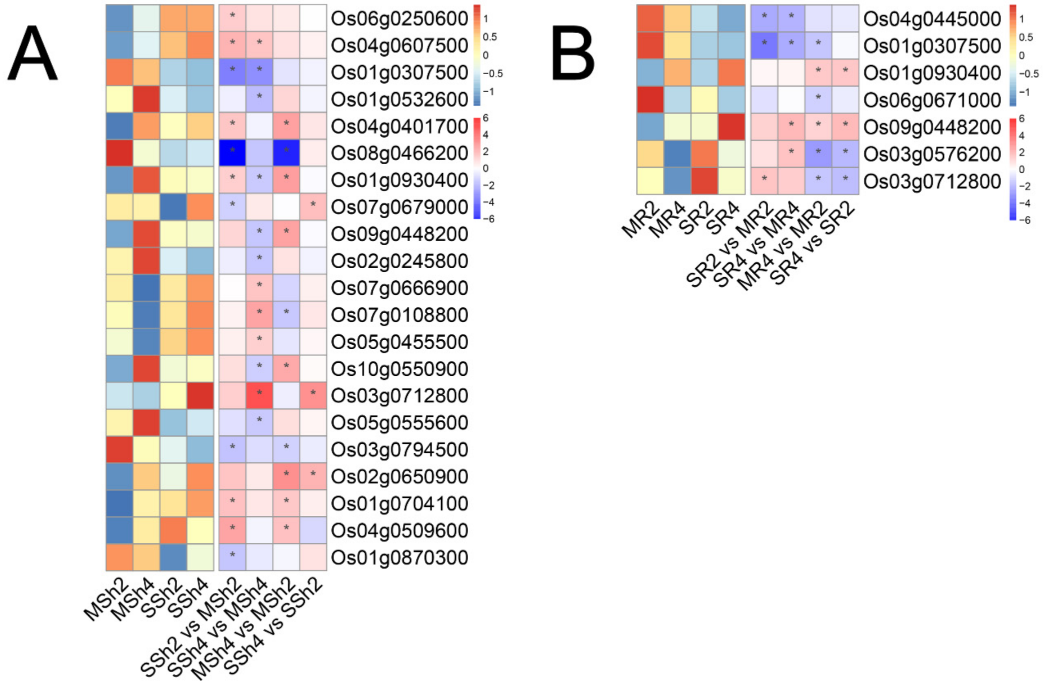

Figure S3. Expression profiles of genes related to ionic transport in shoots and roots of 9311-2x and 9311-4x rice plants under mock and saline stress conditions. Heatmap of differentially expressed genes (DEGs) was generated using MapMan software. Yellow indicates genes expression levels (read_counts per million with log2 value). White indicates the fold change (log2 value) of DEGs. ** $p < 0.01$, * $p < 0.05$.

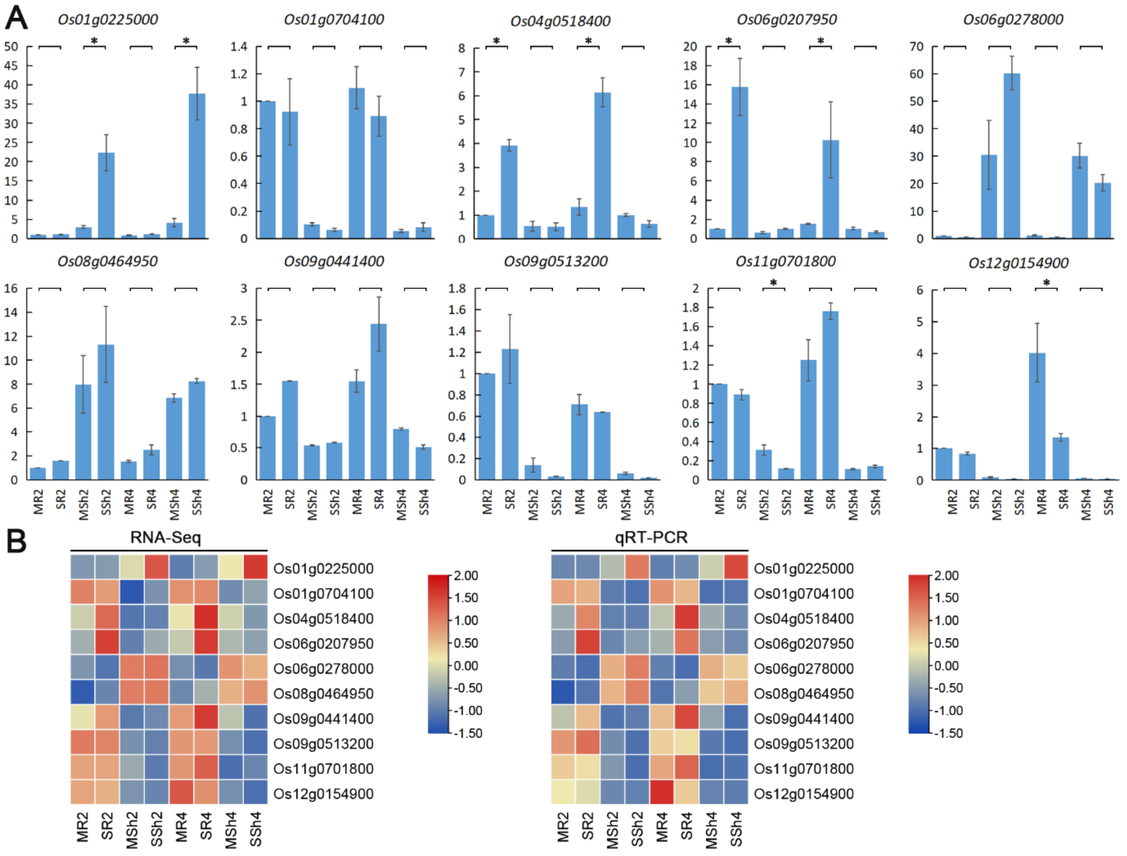

Figure S4. The expression of ten randomly selected genes was analyzed using quantitative reverse transcriptase polymerase chain reaction (qRT-PCR) for the verification of the transcriptome results. * $p < 0.05$.
